# Evaluating penicillin allergy clinical decision-making tools to enhance penicillin allergy delabeling

**DOI:** 10.1016/j.jacig.2026.100719

**Published:** 2026-04-24

**Authors:** Chang Su, Ami P. Belmont, Moeun Son, Jane Liao, Jason Trubiano, Jason Kwah

**Affiliations:** a Section of Rheumatology, Allergy and Immunology, Department of Internal Medicine, Yale University School of Medicine, New Haven, Conn; b Division of Pulmonary, Critical Care, Allergy and Sleep Medicine, University of California San Francisco Medical Center, San Francisco, Calif; c Department of Maternal Fetal Medicine, Yale School of Medicine, New Haven, Conn; d Division of Maternal-Fetal Medicine, Department of Obstetrics and Gynecology, Weill Cornell Medical College, New York, NY; e Division of Allergy and Immunology, Department of Internal Medicine, Montefiore Medical Center, Bronx, NY; f Centre for Antibiotic Allergy and Research, Department of Infectious Diseases, Austin Health, Heidelberg, Australia

**Keywords:** Penicillin allergy, β-lactam allergy, adverse drug reactions, drug allergy, clinical decision-making tools, health outcomes

## Abstract

**Background:**

Patient-reported penicillin allergy is associated with adverse outcomes.

**Objective:**

We aimed to evaluate clinical decision-making tools used to risk-stratify reported penicillin allergies for delabeling.

**Methods:**

Retrospective chart review of patients with reported penicillin allergies who underwent testing from October 9, 2020, to October 8, 2022 was completed at a tertiary referral health care system. Follow-up surveys were conducted to assess penicillin use after testing. The PEN-FAST clinical decision rule, 1-1-1 rule, Siew score, and Vanderbilt criteria were evaluated.

**Results:**

Of the 137 patients who underwent penicillin allergy evaluations, 58 completed follow-up surveys. In all, 48 patients (82.8%) had skin testing, and 46 patients had penicillin challenge following negative skin testing. Of the 10 patients (17.2%) who underwent direct challenge, none had reactions. A total of 4 patients (6.9%) tested positive; 2 had positive skin test results and 2 had negative skin test results but failed oral challenges. In predicting a positive penicillin test, the negative predictive values of the PEN-FAST (score ≤ 2), 1-1-1 rule (score ≤ 2), Siew score (score = 0), and Vanderbilt criteria with only low-risk features were 100% (95% CI = 92.0%-100.0%), 91.3% (95% CI = 72.0%-98.9%), 100.00% (95% CI = 79.4%-100.0%), and 96.6% (95% CI = (82.2%-99.9%), respectively. Of the 54 patients (93.1%) who had negative test results, 21 (38.9%) received subsequent courses of penicillin (to which 18 had no reaction and 3 had delayed rashes); none developed anaphylaxis.

**Conclusions:**

Direct challenge was underutilized in penicillin allergy evaluations even though most patients tested had low-risk histories. The PEN-FAST, 1-1-1 rule, Siew score, and Vanderbilt criteria were all associated with high negative predictive values in our cohort, thus effectively identifying low-risk patients. We advocate the use of these tools to enhance penicillin allergy delabeling.

## Introduction

Patient-reported penicillin allergy is associated with adverse outcomes. Although around 10% of patients have penicillin allergy labels in their medical record, more than 95% of them can tolerate penicillin.^1^^,^^2^ In an effort to improve utilization of first-line antibiotics and public health, various clinical decision-making tools to evaluate penicillin allergy labels have been developed to guide optimal testing strategies and, in some cases, the use of cross-reactive antibiotics (Table I).^1^^,^^3^, ^4^, ^5^, ^6^, ^7^, ^8^, ^9^, ^10^ The PEN-FAST clinical decision rule is a tool that has been externally validated to identify low-risk penicillin allergies that can be tested with a direct penicillin challenge rather than by skin testing followed by penicillin challenge.^4^^,^^7^^,^^8^ The 1-1-1 rule,^3^ Siew score,^5^ and Vanderbilt criteria^6^^,^^9^ are additional tools that use clinical history to risk-stratify patients, but they have not been externally validated. Furthermore, to our knowledge, there has been no direct comparison of the various penicillin allergy clinical decision-making tools. Therefore, we aimed to evaluate and compare the PEN-FAST, 1-1-1 rule, Siew score, and Vanderbilt criteria in identifying cases of IgE-mediated penicillin allergy at a large university-affiliated tertiary referral health care system in the United States.

**Table I:** Clinical decision-making tools for risk-stratifying patients with reported penicillin allergy

Clinical decision-making tools	Scoring criteria	Key performance metrics in prior studies
PEN-FAST	• Reaction within 5 years, 2 points • Anaphylaxis, angioedema, severe cutaneous adverse reaction, 2 points • Treatment required or unknown, 1 point	Total score ≤ 2; NPV = 95%-100%^4^, ^8^
1-1-1 rule	• Rash eruption within 1 hour of penicillin administration, 1 point • Rash development after the first dose, 1 point • Duration of eruption after stopping intake within 1 day, 1 point • Of note, we included patients with any rash, whereas the original rule included only patients with urticaria	Total score = 3; NPV = 80%^3^
Siew score	• Anaphylaxis, 1 point • Known index drug name, 1 point • Reaction within 1 year, 1 point	Total score = 0; NPV = 98.4%^5^
Vanderbilt criteria	• Highest risk: severe delayed symptoms at any point (blistering rash, mucosal involvement, severe rash, and/or immune-mediated organ injury) • Moderate-to-high risk: anaphylaxis • Low risk: inconsistent with high-risk criteria (self-limited cutaneous rash, remote reaction with limited details, urticaria only, reaction was more than 5 years ago, family history)	Low-risk features only; NPV = 99%^6^, ^9^, ^10^

We performed an observational cohort study of nonpregnant patients with reported penicillin allergies who underwent penicillin allergy testing in an allergy/immunology clinic between October 9, 2020, and October 8, 2022. We conducted semistructured phone follow-up surveys to gather additional clinical history regarding past reactions to penicillin and assess penicillin use after testing. Allergy testing included skin prick and intradermal testing followed by oral challenge or direct challenge (DC) without skin testing. The PEN-FAST, 1-1-1 rule, Siew score, and Vanderbilt criteria were compared with testing outcomes. A positive penicillin allergy test result was defined as a positive skin testing result or an immediate allergic reaction within 60 minutes of penicillin challenge.

Sensitivity, specificity, positive predictive value, negative predictive value (NPV), positive likelihood ratio, negative likelihood ratio, and the area under the receiver operating curve (AUC) were calculated for the PEN-FAST (score ≤ 2), 1-1-1 rule (score ≤ 2), Siew score (score = 0), and Vanderbilt criteria with only low-risk features. We followed the protocols as noted in Table I.^3^, ^4^, ^5^, ^6^, ^7^, ^8^, ^9^, ^10^ In accordance with original study protocols, for PEN-FAST, we did not exclude any patients from the analyses if they could not recall reaction details, and for the 1-1-1 rule, Siew score, and Vanderbilt criteria, patients with an incomplete history and therefore incomplete scores were excluded. Of note, the Vanderbilt criteria assigned inadequate history as low-risk and did not exclude any patients with inadequate history in the retrospective validation.^6^^,^^9^^,^^10^ However, we excluded patients who recalled a “severe reaction” or features consistent with a moderate or severe reaction but could not recall the specific symptoms. In addition, because the 1-1-1 rule is based on characteristics of urticaria, patients without any rash were excluded. Data analyses were completed by using Stata, version 19.0 (StataCorp, College Station, Tex).

## Results and discussion

Of the 136 patients with reported penicillin allergies who completed penicillin allergy testing, 58 (42.6%) completed follow-up surveys (Table II). In total, 4 patients (6.9%) had a positive testing result, whereas 54 patients (93.1%) were delabeled. Of the 10 patients (17.2%) who underwent DC, none had reactions. Of the 48 patients (82.8%) who underwent skin testing, 2 (3.4%) had a positive result (1 patient reported a history of hives and full-body itching 7 to 8 days into an amoxicillin course administered 2 years ago; the other patient reported a history of syncope requiring cardiopulmonary resuscitation following a penicillin injection administered 20 to 30 years ago). Of the 46 patients (79.3%) who underwent skin testing followed by oral challenge, 2 (3.4%) failed the oral challenge (1 patient reported a history of anaphylaxis requiring epinephrine more than 30 years ago; 30 minutes after receiving amoxicillin 50 mg in the clinic, the patient developed hives under the left axilla and bilateral thigh pruritus that resolved with oral antihistamines; the other patient reported a history of rash, shortness of breath, wheezing after taking amoxicillin more than 30 years ago; 15 minutes after receiving amoxicillin 50 mg in the clinic, the patient developed cough and wheezing that resolved with an albuterol nebulizer). All of the patients with low-risk PEN-FAST and Siew scores had negative testing results, whereas 2 patients with a low-risk 1-1-1 rule (8%) and 1 patient (2%) with only low-risk features based on the Vanderbilt criteria, respectively, had positive testing results (Table III).

**Table II:** Clinical characteristics of patients who had penicillin allergy evaluation

Characteristics	No. of patients (N = 58)
Age (y), median (IQR)	55 (43-68)
Female sex, no. (%)	43 (74.1)
History of atopy, no. (%)	31 (53.4)
Asthma	18 (31.0)
Allergic rhinitis	20 (34.5)
Atopic dermatitis	1 (1.7)
Hymenoptera venom allergy	1 (1.7)
Food allergy	9 (15.5)
Penicillin skin testing, no. (%)	48 (82.8)
Penicillin challenges, no. (%)	56 (96.6)
Penicillin challenge following skin test	46 (79.3)
DC	10 (17.2)

*IQR*, Interquartile range.

**Table III:** Testing strategy and outcomes of patients with penicillin allergy evaluation according to the PEN-FAST, 1-1-1 rule, Siew score, and Vanderbilt criteria

Clinical decision-making tool	Patients, no. (%) (N = 58)	High-risk score,∗ no. (%)	Low-risk score,∗ no. (%)
PEN-FAST	58 (100)	17 (29.3)	41 (70.7)
DC†	10 (17.2)	1 (1.7)	9 (15.5)
Positive skin test result	2 (3.4)	2 (3.4)	0 (0)
Positive challenge result	2 (3.4)	2 (3.4)	0 (0)
1-1-1 rule	25 (43.1)	2 (8.0)	23 (92.0)
DC†	4 (6.9)	0 (0)	4 (16.0)
Positive skin test result	1 (1.7)	0 (0)	1 (4.0)
Positive challenge result	1 (1.7)	0 (0)	1 (4.0)
Siew score	51 (87.9)	35 (68.6)	16 (31.4)
DC†	8 (13.8)	0 (0)	8 (15.7)
Positive skin test result	2 (3.4)	2 (3.9)	0 (0)
Positive challenge result	2 (3.4)	2 (3.9)	0 (0)
Vanderbilt criteria	49 (84.5)	20 (40.8)	29 (59.2)
DC†	8 (13.8)	1 (2.0)	7 (14.3)
Positive skin test result	2 (3.4)	1 (2.0)	1 (2.0)
Positive challenge result	2 (3.4)	2 (4.0)	0 (0)

∗ The low-risk scores for the clinical decision-making tools are defined as a PEN-FAST score of 2 or lower, 1-1-1 rule score of 2 or lower, Siew score of 0, and Vanderbilt criteria with low-risk features only. The high-risk scores for the clinical decision-making tools are defined as a PEN-FAST score higher than 2, 1-1-1 rule score of 3, Siew score higher than 0, and Vanderbilt criteria with moderate or high-risk features.

† All patients who underwent DC passed the challenge and were delabeled.

In predicting a positive penicillin allergy test result, the NPVs of the PEN-FAST (score ≤ 2), 1-1-1 rule (score ≤ 2), Siew score (score = 0), and Vanderbilt criteria with only low-risk features were 100% (95% CI = 92.0%-100.0%), 91.3% (95% CI = 72.0%-98.9%), 100.00% (95% CI = 79.4%-100.0%), and 96.6% (95% CI = 82.2%-99.9%). Compared with the other tools (AUC = 0.46-0.69), the PEN-FAST captured a high percentage of low-risk patients (70.7%) and demonstrated a high AUC of 0.89 (95% CI = 0.83-0.94) (Table IV).

**Table IV:** Performance of the PEN-FAST, 1-1-1 rule, Siew score, and Vanderbilt criteria in predicting positive penicillin allergy testing

Performance metrics	PEN-FAST score ≤ 2	1-1-1 rule score ≤ 2	Siew score = 0	Vanderbilt criteria with low-risk features only
Sensitivity, % (95% CI)	100 (39.8-100)	0 (0-84.2)	100 (39.8-100)	75.0 (19.4-99.4)
Specificity, % (95% CI)	77.2 (64.2-87.3)	91.3 (72.0-98.9)	34.0 (20.9-49.3)	62.2 (46.5-76.2)
PPV % (95% CI)	23.5 (6.8-49.9)	0 (0-84.2)	11.4 (3.2-26.7)	15.0 (3.2-37.9)
NPV % (95% CI)	100 (92.0-100)	91.3 (72.0-98.9)	100 (79.4-100)	96.6 (82.2-99.9)
PLR (95% CI)	4.38 (2.72-7.07)	0	1.52 (1.23-1.86)	1.99 (1.01-3.91)
NLR (95% CI)	0	1.10 (0.97-1.24)	0	0.40 (0.07-2.23)
AUC (95% CI)	0.89 (0.83-0.94)	0.46 (0.40-0.52)	0.67 (0.60-0.74)	0.69 (0.43-0.94)

*NLR*, Negative likelihood ratio; *PLR*, positive likelihood ratio; *PPV*, positive predictive value.

Of the 54 patients with negative testing (93.1%), 21 (38.8%) received penicillin after testing: 18 of the 21 (85.7%) had no reaction and 3 of the 21 (14.3%) had delayed benign rashes. In all, 33 patients (61.1%) had not received penicillin since testing: 28 of the 33 (84.8%) had not needed penicillin and 5 of the 33 (15.2%) reported during the follow-up survey that they had continued to avoid penicillin despite their negative testing results, delabeling in their medical chart, and their reported understanding of the post-test instructions.

Previous studies have focused on PEN-FAST, a user-friendly tool that has been externally validated.^4^^,^^7^^,^^8^ Our study aimed to evaluate multiple clinical decision-making tools, including the 1-1-1 rule,^3^ Siew score, and Vanderbilt criteria,^6^^,^^9^ which to our knowledge have not previously been externally validated or compared to the PEN-FAST. In our cohort, the PEN-FAST (score ≤ 2), 1-1-1 rule (score ≤ 2), Siew score (score = 0), and Vanderbilt criteria with only low-risk features were all associated with high NPVs (91.6%-100%).

Our data suggest that DC is underutilized in penicillin allergy evaluations both in this study and in current institutional practice. Of 58 patients, only 10 (17.2%) proceeded with DC even though most patients had low-risk histories, and 54 (93.1%) were ultimately delabeled. Given the high NPVs across all tools evaluated in the study, the PEN-FAST, 1-1-1, and Siew scores and the Vanderbilt criteria can all be used to identify and delabel low-risk patients. The PEN-FAST exhibited the highest utility: 100% of the patient histories were complete, all of the low-risk patients passed oral challenge, and PEN-FAST had the highest AUC, potentially demonstrating the best discrimination ability.

In follow-up of the 54 patients with negative testing, 21 (38.9%) received penicillin and none had anaphylaxis, confirming the safety and efficacy of penicillin allergy evaluations. Although most of the patients had either received or felt safe to receive penicillin after delabeling (similar to the results in a prior study^11^), 5 of the 54 patients (9.3%) continued to avoid penicillin.

The limitations of this study include its retrospective, single-center design, small sample size, small number of positive tests, and low follow-up survey response rate, raising concerns about limited statistical power, selection, and referral bias, and ultimately, concerns that these factors could exert disproportionate effects on the statistical results, leading to wide confidence intervals. In addition, formal statistical comparison of the relative performance metrics of the individual clinical decision-making tools could not be completed because the study was not adequately powered for such a comparison, as a result of which the study’s comparative performance and broader applicability were exploratory. Moreover, the exclusion of patients with incomplete clinical histories for the 1-1-1 rule, Siew score, and Vanderbilt criteria (similar to that in the original study protocols) may have resulted in overestimation of the tools’ diagnostic performance and generalizability. Furthermore, false-positive results can occur in both skin testing and oral challenge, particularly in low-risk patients with underlying urticaria or dermatographism. Lastly, the criteria used to select between skin testing followed by challenge versus DC were not standardized or recorded, which could introduce selection bias and unmeasured confounding factors. A strength of this study is that the patient histories were confirmed via phone follow-up surveys and the scoring was adjudicated by internal medicine and allergy/immunology clinicians. Additionally, the testing results were consistent, as all testing was performed at a single allergy/immunology clinic within a large university-affiliated tertiary referral health care system.

To our knowledge, this is the first study to externally validate and compare different clinical decision-making tools to risk-stratify reported penicillin allergy. The PEN-FAST, 1-1-1 rule, Siew score, and Vanderbilt criteria were all associated with high NPVs necessary for a rule-out test in specialist settings, with the PEN-FAST potentially demonstrating the highest utility. Our findings support those of previous studies^3^, ^4^, ^5^^,^^7^^,^^8^ and provide evidence for advocating for the use of these tools to enhance penicillin allergy delabeling. Prospective, multicenter studies of larger cohorts would be beneficial for implementating these tools in a broader clinical setting. Future directions should include quality improvement initiatives to encourage post-testing patient education and DC in contexts beyond outpatient allergy clinics to further improve antibiotic stewardship and patient outcomes.

**Clinical implications**

The PEN-FAST, 1-1-1 rule, Siew score, and Vanderbilt Criteria clinical decision-making tools were all associated with high NPVs and can be used to enhance penicillin allergy delabeling.

## Disclosure statement

Supported by 10.13039/100005326Yale University (Yale Physician Scientist Development Award (to A.P.B.) and the 10.13039/100006108National Center for Advancing Translational Sciences, a component of the 10.13039/100000002National Institutes of Health (Clinical and Translational Science Award UL1 TR001863 [to A.P.B.]). C.S. receives funding from the Allergists’ Foundation Community Grant Program for an unrelated project. The contents of this article are solely the responsibility of the authors and do not necessarily represent the official views of the National Institutes of Health. This research did not receive any specific grant from funding agencies in the public, commercial, or not-for-profit sectors.

Disclosure of potential conflict of interest: The authors declare that they have no relevant conflicts of interest.
